# Salivary Oxidative Stress Increases with the Progression of Chronic Heart Failure

**DOI:** 10.3390/jcm9030769

**Published:** 2020-03-12

**Authors:** Anna Klimiuk, Anna Zalewska, Robert Sawicki, Małgorzata Knapp, Mateusz Maciejczyk

**Affiliations:** 1 Experimental Dentistry Laboratory, Medical University of Bialystok, 24a M. Sklodowskiej-Curie Street, 15-274 Bialystok, Poland; annak04@poczta.onet.pl (A.K.); azalewska426@gmail.com (A.Z.); 2 Department of Cardiology, Medical University of Bialystok, 24a M. Sklodowskiej-Curie Street, 15-274 Bialystok, Poland; r-sawicki@o2.pl (R.S.); malgo33@interia.pl (M.K.); 3 Department of Hygiene, Epidemiology and Ergonomics, Medical University of Bialystok, 2c Mickiewicza Street, 15-233 Bialystok, Poland

**Keywords:** chronic heart failure, saliva, oxidative stress, salivary biomarkers

## Abstract

The aim of the study was to evaluate the rate of reactive oxygen species (ROS) production, antioxidant barrier, and oxidative damage in non-stimulated (NWS) and stimulated (SWS) saliva as well as plasma/erythrocytes of 50 patients with chronic heart failure (HF) divided into the two subgroups: NYHA II (33 patients) and NYHA III (17 patients). The activity of superoxide dismutase and catalase was statistically increased in NWS of HF patients as compared to healthy controls. The free radical formation, total oxidant status, level of uric acid, advanced glycation end products (AGE), advanced oxidation protein products and malondialdehyde was significantly elevated in NWS, SWS, and plasma of NYHA III patients as compared to NYHA II and controls. We were the first to demonstrate that with the progression of HF, disturbances of enzymatic and non-enzymatic antioxidant defense, and oxidative damage to proteins and lipids occur at both central (plasma/erythrocytes) and local (saliva) levels. In the study group, we also observed a decrease in saliva secretion, total salivary protein and salivary amylase activity compared to age- and gender-matched control group, which indicates secretory dysfunction of salivary glands in patients with HF. Salivary AGE may be a potential biomarker in differential diagnosis of HF.

## 1. Introduction

Despite enormous progress made in the diagnosis and treatment of cardiovascular diseases, the incidence of chronic heart failure (HF) is steadily increasing and is the leading cause of death in adults. Thus, HF is not only a major clinical, but also an epidemiological and economic problem [1,2]. It is estimated that HF affects about 1%–2% of the population in developed countries, and in the age group of over 70 the disease already affects every 10^th^ person [3]. According to the current definition, HF is a syndrome of symptoms such as dyspnea, swelling of the lower limbs, and decreased tolerance to physical activity, which may be accompanied by abnormalities in the physical examination (jugular vein dilatation, crackles over the lungs, peripheral swelling) [4]. Differentiation of HF patients based on the left ventricular ejection fraction (LVEF) is essential due to other underlying etiologies, demographic differences, co-morbidities, and responses to applied treatment [3]. Thus, we distinguish patients with normal LVEF (typically considered as ≥ 50%; HF with preserved EF (HFpEF)) as well as reduced LVEF (typically considered as < 40%; HF with reduced EF (HFrEF)) [3]. The cause of these ailments in HFrEF is a disturbed structure and/or function of the heart, which results in decreased cardiac output and increased intracardiac pressure at rest and/or during physical activity [1,2,4]. Despite varied pathogenesis (cardiac disease, pre- and post-inflammatory stress disorder, and heart arrhythmia), HF leads to impaired supply of oxygen to the body tissues depending on the current metabolic demand [1,2,4]. Importantly, many patients with HF have a history of myocardial infarction or revascularization [3].

Studies on HF indicate that an important role in its pathogenesis is played by oxidative stress [5,6,7]. This process creates an imbalance between the production of reactive oxygen species (ROS) and antioxidative defense, which leads to oxidative damage to proteins, lipids, and nucleic acids, causing structural damage to cells as well as disturbances in tissue integrity [8]. Indeed, the role of oxidative stress has been confirmed in the course of: Endothelial dysfunction, interstitial fibrosis of the heart, cardiomyocyte hypertrophy, and heart extracellular matrix remodeling [5,7,9,10]. Moreover, it has been demonstrated that in the early stages of HF, increased activity of antioxidant enzymes is the basic compensatory mechanism aimed at maintaining adequate blood flow and perfusion of tissues. However, HF progression leads to the depletion of antioxidant reserves and, along with increased free radical production, contributes to further progression of the disease [5,7,9,10].

Increasing attention is being paid to the use of saliva in biomedical diagnostics [11]. Unlike other bioliquids, saliva is a non-infectious laboratory material collected in a manner that is non-invasive, inexpensive, and stress-free for the patient. It is also worth noting that the composition of saliva reflects dynamic changes in the body, which makes this fluid an excellent tool for determining clinically useful biomarkers [12,13,14,15]. Salivary redox biomarkers are used in the diagnosis of diseases of affluence (obesity, insulin resistance, diabetes, chronic kidney disease) [16,17,18], neurodegenerative diseases (Alzheimer’s disease, Parkinson’s disease, dementia) [19,20], or cancer (colorectal, breast, ovarian cancer) [21,22]. However, salivary redox homeostasis has not been evaluated yet in patients with chronic heart failure. Considering that oxidative stress plays a key role in HF pathogenesis [5,6,7], secretory dysfunction of salivary glands may also occur in HF patients, similarly to other diseases with proven etiology of oxidative stress. Indeed, a relationship between oxidative stress and impaired salivary gland function has been demonstrated in obesity [23,24], insulin resistance [25,26], type 1 diabetes [27], and psoriasis [28]. Therefore, it is necessary to assess the salivary redox parameters in patients with HF, as well as the secretory function of the salivary glands. Because redox homeostasis cannot be characterized by a single biomarker, the aim of our work was to compare the rate of ROS production, enzymatic and non-enzymatic antioxidant barriers, and oxidative damage to proteins and lipids in non-stimulated and stimulated saliva, as well as plasma/erythrocytes of patients with chronic heart failure and healthy controls.

## 2. Materials and Methods

### 2.1. Ethical Issues

The study was approved by the Bioethics Committee of the Medical University of Bialystok, Poland (permission number R-I-002/75/2016). After a detailed explanation of the purpose of the study and presentation of possible risks, all persons involved agreed in writing to participate in the experiment.

### 2.2. Patients

The study group consisted of 50 patients (7 women and 43 men) treated in the Department of Cardiology with Intensive Cardiac Supervision of the Medical University of Bialystok Clinical Hospital from May 2018 to April 2019. In all patients, medical history, physical examination, resting ECG, echocardiography, chest X-ray as well as blood laboratory tests (blood count, electrolytes, glucose, hepatic and renal indicators, thyroid hormones, vitamin D, NT-proBNP, and BNP) were performed. The study included hemodynamically stable patients with chronic heart failure, qualified for the implantation of automatic cardioverter defibrillator [3]. The criterion of classification for the procedure was left ventricular ejection fraction (LVEF) ≤ 35%. Based on the classification of heart failure according to the New York Heart Association [3], the study group was divided into two subgroups of patients:

- NYHA II (*n* = 33)—patients with slight physical activity limitations—with no symptoms at rest, but in whom normal activity causes fatigue, palpitations, or shortness of breath;
- NYHA III (*n* = 17)—patients with significant physical activity limitations—with no symptoms at rest, but in whom activity lower than normal provokes symptoms.

All patients were qualified for examinations by the same experienced cardiologist (M. K.), based on the inclusion and exclusion criteria. In the study group, primary prevention has been applied to reduce the risk of sudden death and total mortality in patients with symptomatic HF (NYHA class II-III) and LVEF ≤ 35% (despite at least 3 months of optimal pharmacotherapy), who are expected to survive in good condition for more than one year, and have ischemic heart disease or dilated cardiomyopathy [3]. After classification for the study, all patients underwent coronary angiography (before the cardioverter implantation procedure).

The control group, matched to the study group by gender and age, consisted of 50 generally healthy patients (7 women and 43 men), enrolled for follow-up visits at the Restorative Dentistry Clinic of the Specialist Dental Infirmary of the Medical University of Bialystok from September 2018 to June 2019.

Only patients with a body mass index (BMI) within the range of 18.5–24.5 were qualified to both the study and control group. The exclusion criterion in both groups was the occurrence of chronic systemic and autoimmune diseases (type 1 diabetes, Sjogren’s syndrome, rheumatoid arthritis, psoriasis), lung, thyroid, liver, kidney, gastrointestinal tract diseases, infectious diseases (HCV, HBV and HIV infections), and immunity disorders. Moreover, the study did not allow patients with periodontal diseases, smokers, alcohol drinkers, or patients taking antibiotics, non-steroidal anti-inflammatory drugs, glucocorticosteroids, vitamins, and dietary supplements for the last 3 months. Detailed patient characteristics are presented in Table 1.

### 2.3. Research Material

The research material consisted of venous blood as well as non-stimulated whole saliva (NWS) and stimulated whole saliva (SWS) collected from patients via the spitting method. Material for all examinations was collected before implantation of an automatic cardioverter defibrillator or a resynchronization system.

### 2.4. Blood Collection

Upon fasting, after an overnight rest, venous blood (10 mL) was collected using S-Monovette^®^ K3 EDTA blood collection system (Sarstedt). Blood samples were centrifuged at 1500× g (10 min, +4 °C; MPW 351, MPW Med. Instruments, Warsaw, Poland). In order for the sample to qualify for the study, it had to reveal no hemolysis. The upper layer, i.e., the plasma, was collected and erythrocytes were rinsed three times with a cold solution of 0.9% NaCl and hemolyzed by adding 9 volumes of cold 50 mM phosphate buffer with pH 7.4 (*v*/*v*) [29]. Butylated hydroxytoluene (BHT) antioxidant was added to the samples to protect them against oxidation processes (5 μL 0.5 M BHT in acetonitrile per 0.5 mL plasma/erythrocytes) [17]. The samples were stored at -80 °C (not longer than half a year).

### 2.5. Saliva Collection

Saliva was collected in the morning between 8 a.m. and 10 a.m. to minimize the effect of circadian rhythm on secretion. The procedure was performed in a room where patients were not disturbed, and were rested in a sitting position with the head slightly inclined downwards, with minimized facial and lip movements. After rinsing the mouth three times with distilled water at room temperature, the patients spat out the saliva accumulated at the bottom of the mouth into a sterile Falcon-type tube placed in an ice container. Saliva collected during the first minute was discarded [18,30]. Two hours prior to saliva collection, the study/control group had not consumed any food or drinks (excluding clean water) and refrained from performing oral hygiene procedures. Moreover, at least 8 h before saliva collection, the subjects had not taken any medications. The time of NWS collection was 10 min. After a 5-min break, SWS was collected for 5 min to a maximum volume of 5 mL, upon sprinkling 10 µL of 2% citric acid at the tip of the tongue every 30 s [18,30]. The collected saliva was immediately centrifuged (3000× g, 20 min, +4 °C) [17]. BHT (5 μL 0.5 M BHT in acetonitrile per 0.5 mL supernatant saliva) was added to the obtained supernatants to protect them from oxidation, and then they were frozen to −80 °C (and stored no longer than 6 months) [17].

### 2.6. Dental Examination

The dental examination was conducted in accordance with the World Health Organization criteria in artificial lighting, using a mirror, an explorer, and a periodontal probe [31]. Every examination was performed by the same dentist (A. K.), immediately after the non-stimulated and stimulated saliva collection. Decay, missing, filled teeth (DMFT), Papilla Bleeding Index (PBI), Gingival Index (GI), and the occurrence of carious lesions of root cement (CR) were determined. The DMFT index is the sum of teeth with caries (D), teeth extracted due to caries (M), and teeth filled because of caries (F). The PBI showed the intensity of bleeding from the gingival papilla after probing [32]. GI criteria include qualitative changes in the gingiva [33]. Inter-rater agreements were assessed in 30 patients. The reliability for all the parameters was > 0.98.

### 2.7. Salivary Protein

The salivary protein concentration was determined by spectrophotometric method using bicinchoninic acid (BCA) [34]. Under the influence of protein in alkaline environment, BCA forms a stable complex with Cu^+^ copper ions, that reaches its maximum absorbance at 562 nm wavelength. A commercial set of reagents (Thermo Scientific PIERCE BCA Protein Assay; Rockford, USA) was used to evaluate the salivary protein.

### 2.8. Salivary Amylase

The salivary amylase activity (EC 3.2.1.1) was determined spectrophotometrically using an alkaline solution of 3,5-dinitrosalicylic acid (DNS). We measured the absorbance of samples at 540 nm accompanying the increase in the concentration of reducing sugars released during the hydrolysis of starch catalyzed by salivary amylase. Salivary amylase activity was determined in duplicate samples and expressed in µmol/mg protein.

### 2.9. Redox Assays

Antioxidant enzymes were evaluated in erythrocytes, NWS, and SWS, while non-enzymatic antioxidants were evaluated by redox status, protein/lipid oxidation products in the plasma, NWS, and SWS [17,23,29,35,36].

### 2.10. Antioxidant Barrier

The activity of superoxide dismutase (SOD; E.C. 1.15.1.1) was determined by the Misra and Fridrovich method [37]. The test sample was heated to 37 °C, then adrenaline solution was added, and changes in absorbance at 480 nm wavelength were monitored. It was assumed that 1 unit of SOD corresponds to 50% inhibition of adrenaline self-oxidation to adrenochrome. SOD activity was expressed in mU/mg protein.

Catalase activity (CAT; EC 1.11.1.6) was determined by the Aebi spectrophotometric method [38]. The rate of hydrogen peroxide decomposition in the presence of a blind sample was assayed by measuring the decrease in absorbance at 240 nm wavelength. We defined 1 unit of CAT as the amount of the enzyme needed to decompose 1 mmol of hydrogen peroxide per minute. CAT activity was expressed in nmol H_2_O_2_/min/mg protein.

The activity of salivary peroxidase (Px; EC 1.11.1.7) was determined spectrophotometrically according to the method by Mansson-Rahemtulla et al. [39]. The method involves the reduction of 5,5′-dithiobis-(2-nitrobenzoic acid) (DTNB) to 5′-thio-2-nitrobenzoic acid (TNB). Changes in the absorbance of TNB, depending on Px activity, were measured at λ = 412 nm. The activity of Px was expressed in mU/mg protein.

The activity of glutathione peroxidase (GPx; EC 1.11.1.9) in erythrocytes was determined spectrophotometrically based on reduced nicotinamide adenine dinucleotide (NADPH) conversion to nicotinamide adenine dinucleotide cation (NADP^+^) [40]. The absorbance of the samples was measured at 340 nm wavelength. It was assumed that 1 GPx unit catalyzes the oxidation of 1 mmol NADPH per minute. The activity was expressed in mU/mg protein.

The concentration of reduced glutathione (GSH) was determined spectrophotometrically based on the reduction of DTNB to 2-nitro-5-mercaptobenzoic acid under the influence of GSH contained in the tested sample [41]. Absorbance of the samples was measured at 412 nm. The GSH concentration was calculated from the standard curve for GSH solutions and presented as µg/mg protein.

Uric acid (UA) concentration was determined spectrophotometrically using the ability of 2,4,6-tris(2-pyridyl)-s-triazine to form a blue complex with iron ions in the presence of UA. Absorbance of the samples was measured at 490 nm. We used a commercial set of reagents (QuantiChromTMUric Acid Assay Kit DIUA-250; BioAssay Systems, Hayward, CA, USA). UA concentration was expressed in ng/mg protein (NWS, SWS) and µg/mg protein (plasma).

The activity of antioxidant enzymes was determined in NWS, SWS, and erythrocytes, and the concentrations of non-enzymatic antioxidants were determined in NWS, SWS, and plasma. All determinations were performed in duplicate samples, and the absorbance of the samples was measured with the Infinite M200 PRO Multimode Microplate Reader (Tecan).

### 2.11. Redox Status and ROS Production

Total antioxidant capacity (TAC) was determined spectrophotometrically according to Erel [42]. Changes in absorbance of ABTS^*+^ (3-ethylbenzothiazoline-6-sulfonic acid radical cation) solution were measured at 660 nm. TAC was calculated from the standard curve for Trolox (6-hydroxy-2,5,7,8-tetramethylchroman-2-carboxylic acid). The intensity of ABTS^*+^ color is proportional to the content of antioxidants in the tested sample. The TAC level was expressed in Trolox µmol/mg protein.

Total oxidant status (TOS) was determined spectrophotometrically according to Erel [43]. In the presence of oxidants contained in the sample, iron Fe^2+^ ions were oxidized to Fe^3+^ irons which then formed a colored complex with xylenol orange. Absorbance of the created complex was measured at the TOS level calculated from the standard curve for hydrogen peroxide and presented as nmol H_2_O_2_/min/mg protein.

The oxidative stress index (OSI) was calculated by dividing TOS by TAC, and expressed in % [28].

The rate of ROS production in saliva was determined by chemiluminescence immediately after saliva collection [44]. Luminol was used as an electron acceptor, and hydrogen peroxide as a positive control. Chemiluminescence of samples was measured in 96-well microplates with black bottoms. ROS production rate was expressed as nmol O_2-_/min/mg protein.

TAC, TOS, and OSI levels were evaluated in NWS, SWS, and blood plasma, while the rate of ROS production was determined only in saliva samples. All determinations were performed in duplicate samples (TOS in triplicate samples) and the absorbance/chemiluminescence of samples was measured with Infinite M200 PRO Multimode Microplate Reader (Tecan).

### 2.12. Protein and Lipid Oxidation Products

The content of advanced glycation end products (AGE) was determined spectrofluorimetrically according to Kalousová et al. [45]. The samples were diluted in 0.02 M PBS buffer (1:5, *v*/*v*), and fluorescence was measured at 350 nm excitation wavelength and 440 nm emission. AGE content was expressed in arbitrary fluorescence units (AFU) per mg of total protein.

The concentration of advanced oxidation protein products (AOPP) was determined spectrophotometrically according to Kalousová et al. [45]. The samples were diluted in 0.02 M PBS buffer (1:5, *v*/*v*) and the oxidative capacity of iodine ions was evaluated at 340 nm wavelength. The concentration was expressed in nmol/mg protein.

The concentration of malondialdehyde (MDA) was determined spectrophotometrically using thiobarbituric acid (TBA) [46]. The absorbance of the samples was measured at 535 nm, and 1,1,3,3-tetraethoxypropane was used as a standard. The concentration was expressed in µmol/mg protein.

The content of oxidative products of protein and lipid modifications was determined in NWS, SWS, and blood plasma. All assays were performed in duplicate samples and the absorbance/fluorescence of the samples was measured with Infinite M200 PRO Multimode Microplate Reader (Tecan).

### 2.13. Statistical Analysis

Statistical analysis of the results was performed using GraphPad Prism 7.0 for MacOS (GraphPad Software, La Jolla, USA). The D’Agostino-Pearson test and Shapiro–Wilk test were used to evaluate the distribution of the results. The homogeneity of variance was checked by Levine’s test. The groups were compared using ANOVA analysis of variance and the Tukey’s test, and where no distribution normality was obtained, ANOVA Kruskal–Wallis and Dunn’s tests were used. Multiplicity adjusted *p* value was also calculated. Since most of the data showed a normal distribution, the results were presented as mean ± SEM. Correlations between redox biomarkers were assessed based on the Pearson correlation coefficient. The analysis of diagnostic usefulness of redox biomarkers was assessed by receiver operating characteristic (ROC) analysis. Statistically significant value was *p* ≤ 0.05.

The number of patients was determined based on the previous pilot study, and the power of the test was assumed as 0.9.

## 3. Results

### 3.1. Dental Examination and Salivary Gland Function

The secretory activity of salivary glands was analyzed based on salivary flow rate and evaluation of total protein concentration as well as α-amylase activity in saliva. We observed a significantly lower flow of NWS and SWS in patients with NYHA II and NYHA III compared to the controls (in all cases *p* ˂ 0.001). The total protein content was considerably lower only in NWS in patients from both groups compared to the control group *(p* = 0.04, *p* ˂ 0.001, respectively). The salivary amylase activity was considerably lower in NWS of NYHA II and NYHA III patients (*p* < 0.001, *p* = 0.009, respectively) and SWS of NYHA II patients (*p* = 0.02) compared to the controls. However, no significant differences in oral hygiene as well as gum and periodontal condition (DMFT, PBI, GI, and CR) were found in the study group compared to the controls (Table 2).

### 3.2. Salivary Antioxidants

The antioxidant barrier was evaluated by measuring the activity of antioxidant enzymes (SOD, CAT, Px, GPx) as well as the concentration of non-enzymatic antioxidants (GSH, UA). In NWS, the activity of SOD (*p* ˂ 0.001, *p* = 0.009, respectively), CAT (in both cases *p* ˂ 0.001), and UA (*p* = 0.03, *p* ˂ 0.001, respectively) was significantly higher, while GSH content (in both cases *p* ˂ 0.001) was lower in NYHA II and NYHA III groups compared to the control group. The activity of Px (*p* = 0.02) was considerably lower in NWS of NYHA II patients than in healthy controls. Within the study group, only the concentration of UA was statistically significantly higher in NWS of NYHA II group compared to NYHA III (*p* = 0.02).

**Figure 1:**
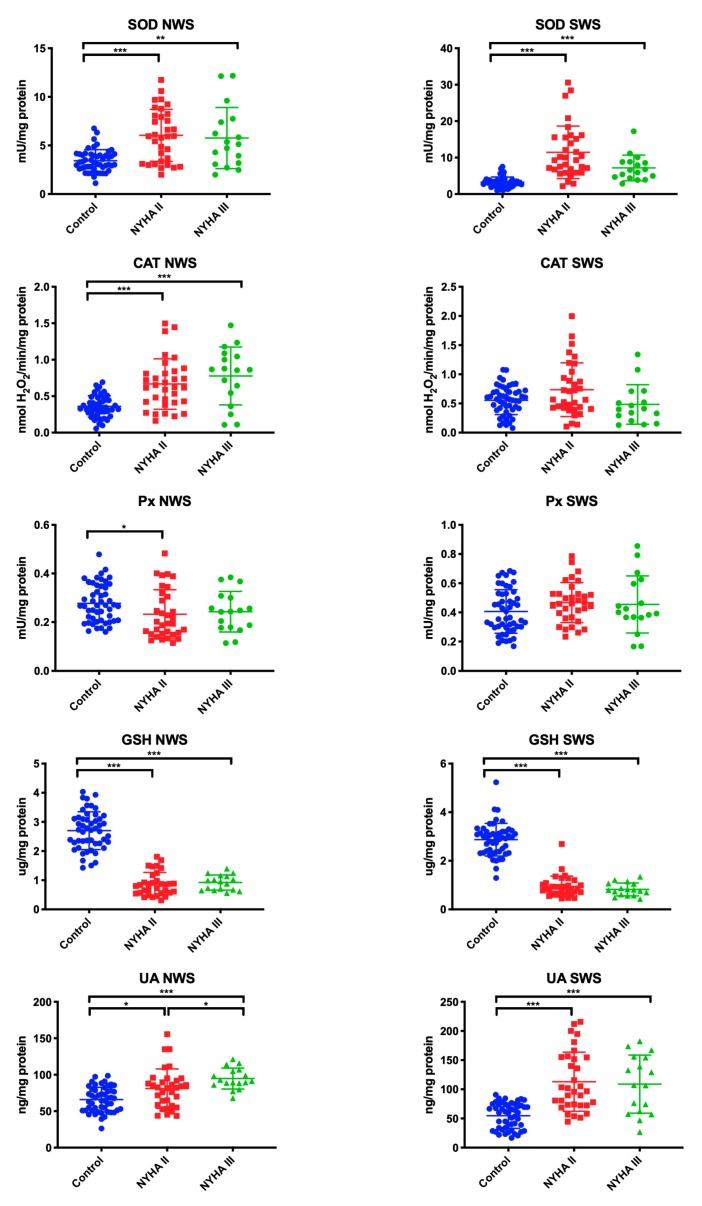
Enzymatic and non-enzymatic antioxidants in non-stimulated and stimulated of HF patients and the control group. Abbreviations: CAT—catalase; GPx—glutathione peroxidase; GSH—reduced glutathione; NWS—non-stimulated whole saliva; NYHA II—class II in the New York Heart Association (NYHA) classification of the heart failure; NYHA III—class III in the New York Heart Association (NYHA) classification of the heart failure; Px—salivary peroxidase; SOD—superoxide dismutase-1; SWS—stimulated whole saliva; UA—uric acid. Differences statistically important at: *p* *< 0.05, **< 0.01, ***< 0.001.

In SWS, the activity of SOD *(p* ˂ 0.001 in both groups) and UA (*p* ˂ 0.001 in both cases) was statistically significantly higher in NYHA II and NYHA III patients compared to the control group. On the other hand, GSH concentration was statistically considerably lower in patients with NYHA II and NYHA III compared to the controls (*p* ˂ 0.001 in both cases), similarly to NWS (Figure 1).

### 3.3. Salivary Total Antioxidant/Oxidant Status

The total redox status was assessed by measuring TAC, TOS, and OSI (TAC/TOS ratio), while the rate of ROS production in saliva was determined by chemiluminescence. In NWS, the mean value of TOS, OSI, and ROS was statistically significantly higher in patients with NYHA II and NYHA III compared to the control group, and in case of ROS—also within the study group (higher in NYHA III). TAC determinations revealed that the values in patients from both study groups were considerably lower than in the control group (in all cases *p* ˂ 0.001).

**Figure 2:**
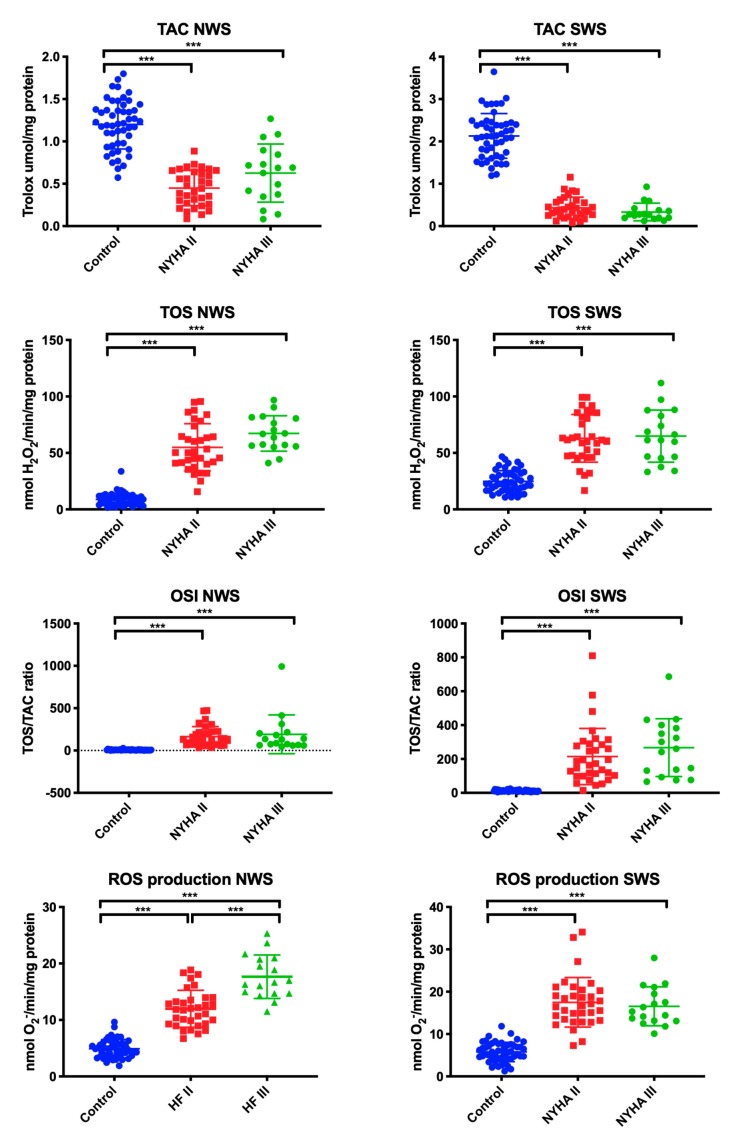
Total antioxidant/oxidant status in non-stimulated and stimulated saliva of HF patients and the control group. Abbreviations: NWS—non-stimulated whole saliva; NYHA II—class II in the New York Heart Association (NYHA) classification of the heart failure; NYHA III—class III in the New York Heart Association (NYHA) classification of the heart failure; OSI—oxidative stress index; SWS—stimulated whole saliva; TAC—total antioxidant capacity; TOS—total oxidant status. Differences statistically important at: *p* **< 0.01, ***< 0.001.

In SWS, the mean value of TOS, OSI, and ROS was significantly higher in patients with NYHA II and NYHA III compared to the control group, while the mean value of TAC was lower (in all cases *p* ˂ 0.001) (Figure 2).

### 3.4. Salivary Oxidative Damage Products

Oxidative stress was assessed by measuring the oxidative damage to proteins (AGE, AOPP) and lipids (MDA). We observed a significant increase in AGE, AOPP, and MDA concentrations in patients with NYHA II and NYHA III in comparison with the control group (in all cases *p* ˂ 0.001) and within the study group (*p* = 0.04, *p* = 0.03, *p* = 0.02, respectively).

**Figure 3:**
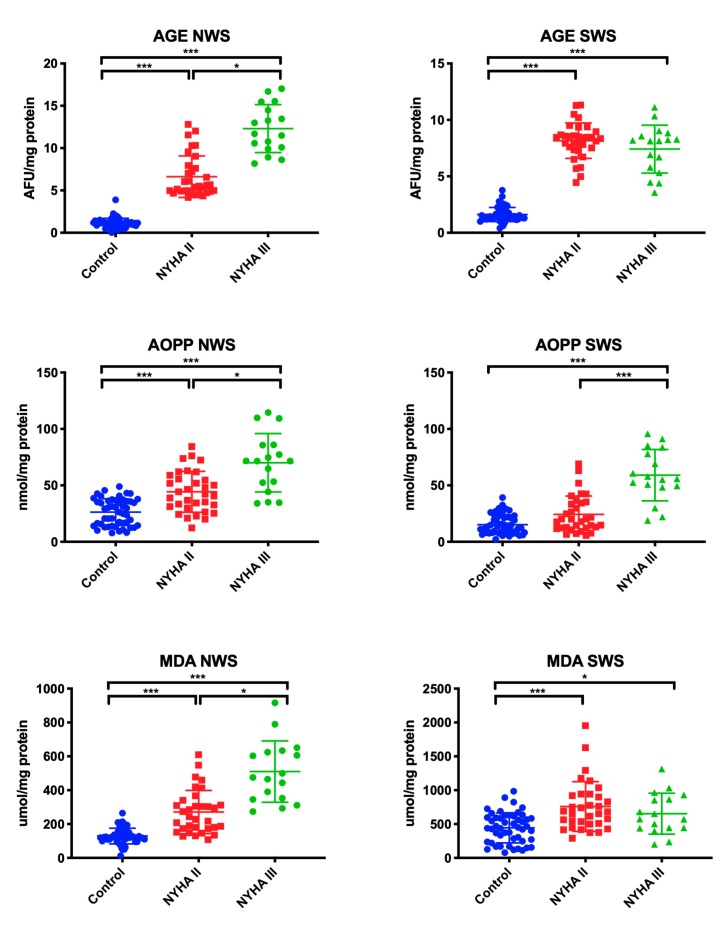
Oxidative damage to proteins and lipids in non-stimulated and stimulated saliva of HF patients and the control group. Abbreviations: AGE—advanced glycation end products; AOPP—advanced oxidation protein products; MDA—malondialdehyde; NWS—non-stimulated whole saliva; NYHA II—class II in the New York Heart Association (NYHA) classification of the heart failure; NYHA III—class III in the New York Heart Association (NYHA) classification of the heart failure; SWS—stimulated whole saliva. Differences statistically important at: *p* *< 0.05, ***< 0.001.

In SWS, levels of AGE and MDA were considerably higher in NYHA II and NYHA III patients compared to healthy controls (*p* ˂ 0.001, *p* ˂ 0.001, *p* ˂ 0.001, *p* = 0.05, respectively). AOPP concentration was statistically significantly higher in patients with NYHA III compared to the control group (*p* ˂ 0.001), and in patients with NYHA III compared to those with NYHA II (*p* ˂ 0.001) (Figure 3).

### 3.5. Erythrocytes/Plasma Redox Biomarkers

**Figure 4:**
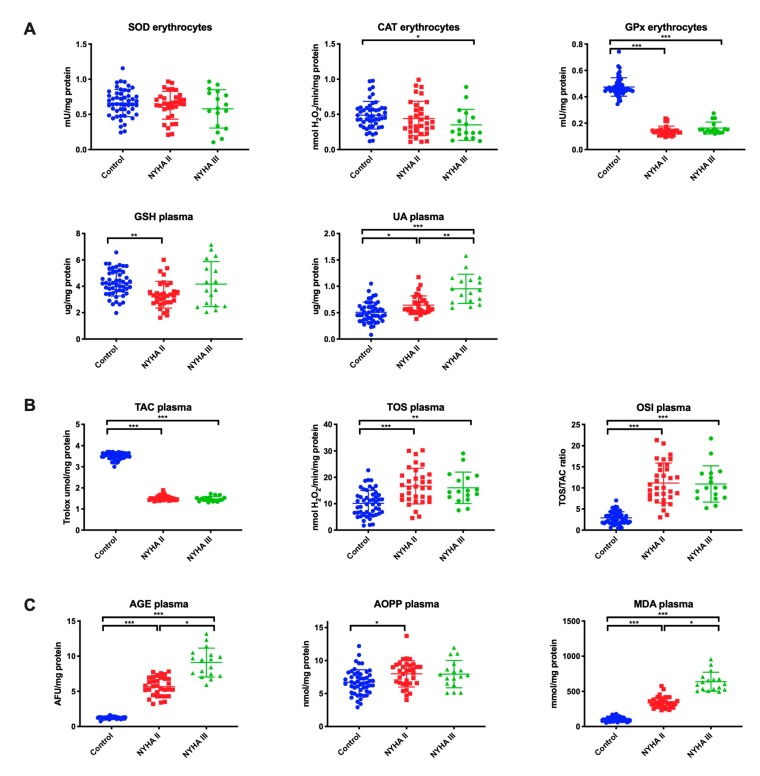
Antioxidant barrier (**A**), redox status (**B**), and oxidative damage to proteins and lipids (**C**) in the erythrocytes/plasma of HF patients and the control group. Abbreviations: AGE—advanced glycation end products; AOPP—advanced oxidation protein products; CAT—catalase; GPx—glutathione peroxidase; GSH—reduced glutathione; MDA—malondialdehyde; NYHA II—class II in the New York Heart Association (NYHA) classification of the heart failure; NYHA III—class III in the New York Heart Association (NYHA) classification of the heart failure; OSI—oxidative stress index; Px—salivary peroxidase; SOD—superoxide dismutase-1; TAC—total antioxidant capacity; TOS—total oxidant status; UA—uric acid. Differences statistically important at: *p* *< 0.05, ***< 0.001.

Antioxidant barrier (A), redox status (B), and oxidative damage to proteins and lipids (C) in the erythrocytes/plasma of HF patients and the control group are presented in Figure 4.

GPx activity (*p* ˂ 0.001 in both study groups) in erythrocytes was significantly lower in patients with NYHA II and NYHA III compared to the control group, whereas CAT activity (*p* = 0.03) in erythrocytes was lower only in NYHA III patients, and GSH concentration (*p* = 0.002) in plasma—only in NYHA II patients. UA concentration was considerably higher both in NYHA II and NYHA III patients compared to the control and within the study group (*p* = 0.02, *p* ˂ 0.001, *p* = 0.003, respectively) (Figure 4A).

TOS and OSI in plasma of NYHA II and NYHA III patients reached statistically significantly higher levels compared to the control group, while the level of TAC was lower (in all cases *p* ˂ 0.001) (Figure 4B).

The plasma concentrations of AGE and MDA were considerably higher in patients with NYHA II and NYHA III than in the control group (*p* ˂ 0.001, *p* ˂ 0.001, *p* ˂ 0.001, *p* = 0.02, respectively) and in patients with NYHA III compared to NYHA II (*p* = 0.04, *p* = 0.02, respectively). The content of AOPP was statistically significantly higher in NYHA II patients compared to the control group (*p* = 0.01) (Figure 4C).

### 3.6. Correlations

In the group of patients with chronic heart failure, we showed a positive correlation between SOD activity in saliva (both NWS and SWS) and ROS production rate (r = 0.825, *p* <0.0001 and r = 0.864, *p* <0.0001, respectively) and negative correlation between Px activity and ROS formation in NWS (r = −0.836, *p* <0.0001). In NWS and SWS we also showed a negative correlation between GSH and AGE concentration (r = −0.858, *p* <0.0001 and r = −0.873, *p* <0.0001, respectively) and GSH and AOPP (r = −0.814, *p* <0.0001 and r = −0.768, *p* <0.0001, respectively).

**Figure 5:**
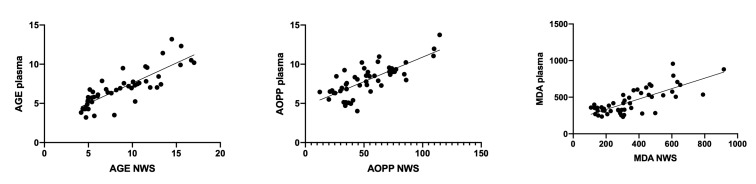
Correlations between salivary and plasma AGE, AOPP, and MDA in patients with heart failure. Abbreviations: AGE—advanced glycation end products; AOPP—advanced oxidation protein products; MDA—malondialdehyde; NWS—non-stimulated whole saliva.

We also observed a negative correlation between NWS flow rate and AGE and MDA content (r = −0.846, *p* <0.0001 and r = −0.847, *p* <0.0001, respectively) and a positive relationship between ROS production rate in SWS and AOPP concentration (r = 0.892, *p* <0.0001). The concentration of AGE (r = 0.84, *p* <0.001), AOPP (r = 0.756, *p* <0.001), and MDA (r = 0.76, *p* <0.001) in NWS correlated positively with their plasma content (Figure 5).

**Figure 6:**
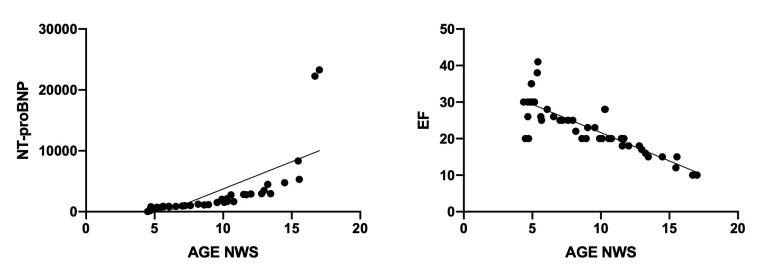
Correlations between salivary AGE content, serum NT-proBNP, and cardiac ejection fraction in patients with heart failure. Abbreviations: AGE—advanced glycation end products; EF—ejection fraction; NT-proBNP—N-terminal fragment of prohormone B-type natriuretic peptide; NWS—non-stimulated whole saliva.

Interestingly, we also observed positive correlation between AGE content in NWS and serum NT-proBNP (r = 0.711, *p* <0.0001) and the negative relationship between the AGE content and cardiac ejection fraction (r = −0.832, *p* <0.0001) (Figure 6).

### 3.7. ROC Analysis

**Figure 7:**
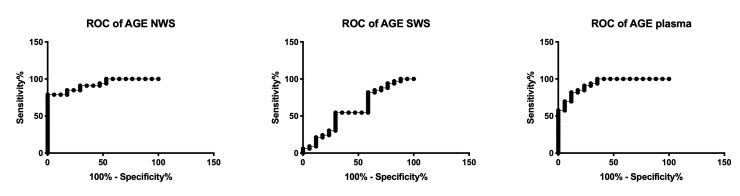
Receiver operating characteristic (ROC) analysis of AGE in non-stimulated and stimulated saliva as well as plasma of NYHA II and NYHA III patients. Abbreviations: AGE—advanced glycation end products; NWS—non-stimulated whole saliva; NYHA II—class II in the New York Heart Association (NYHA) classification of the heart failure; NYHA III—class III in the New York Heart Association (NYHA) classification of the heart failure; SWS—stimulated whole saliva.

The results of ROC analysis for redox biomarkers are presented in Table 3. UA concentration in NWS and plasma, the rate of ROS production in NWS, and the concentration of all oxidative damage products in NWS (AGE, AOPP, MDA) significantly differentiate NYHA II patients compared to NYHA III. Moreover, the assessment of AOPP concentration in SWS, as well as AGE and MDA in plasma, has a high diagnostic value in differentiating the progression of chronic heart failure (Table 3, Figure 7).

## 4. Discussion

In the presented experiment, we studied the salivary gland function as well as redox homeostasis in the saliva and blood of HF patients. Generally, the progression of HF increases oxidative stress not only at the central level (blood), but also in the NWS and SWS. Additionally, in patients with HF, there is a dysfunction of salivary glands and abnormal protein secretion to saliva. Our study was the first to characterize salivary redox profile in HF patients and evaluate the clinical usefulness of salivary biomarkers in the differential diagnosis of heart failure.

HF is a clinical disease entity of multifactorial etiology connected with hypercholesterolemia, hypertension, diabetes, smoking, unbalanced diet, and sedentary lifestyle [1,2,4]. Oxidative stress is considered both the primary and secondary cause of HF, similarly to many other systemic diseases [5,6,7]. Indeed, the key contribution of oxidative stress in HF has been demonstrated in the pathogenesis of genetic, neurodegenerative, neoplastic, and metabolic diseases. Therefore, redox biomarkers are gaining increasing popularity in clinical laboratory diagnostics [47,48,49,50,51].

Direct analysis of reactive oxygen species in the body is a very difficult task. Each of the oxidants causes specific cellular (protein/lipid) modifications. Therefore, a number of different biomarkers must be used to assess redox homeostasis [52]. In our study, we evaluated enzymatic (SOD, CAT, Px) and non-enzymatic (GSH, UA) antioxidant systems, total redox status (TAC, TOS, OSI), as well as protein (AGE, AOPP) and lipid (MDA) oxidative damage products.

An important factor influencing therapeutic success is non-invasive collection of the material for examinations, which reduces patients’ anxiety and contributes to a greater desire to monitor one’s health status and diagnose the disease at its early stage. An interesting alternative to blood—which is commonly used in diagnostics—is saliva. This bioliquid is produced by large salivary glands (parotid, submandibular and sublingual) as well as numerous smaller salivary glands spread throughout the oral cavity. Saliva is blood plasma filtrate, and consists of electrolytes, proteins (immunoglobulins, enzymes, mucins), hormones, and vitamins. It is also a rich source of antioxidants (Px, CAT, SOD, GSH, UA) [14,53].

Antioxidants play an important role in preventing oxidative damage to biomolecules. In the group of HF patients, we observed a significant increase in the activity of salivary antioxidant enzymes (SOD in NWS and SWS, CAT in NWS), and increased concentration of UA (in NWS, SWS, and plasma) vs. the control, which can be considered an adaptive response of the body to intensified production of free radicals. It is also confirmed by positive correlation between SOD activity in NWS and SWS and free radical production rate. It is well known that the main source of ROS in HF is the excessive activation of the renin-angiotensin-aldosterone system [5,54], which stimulates the NFkB signaling pathway and thus boosts production of proinflammatory cytokines, but also the activity of NADPH oxidase (NOX), which is the primary source of free radicals in the cell [8]. This is also evidenced by the negative correlation between Px activity in NWS and ROS production rate. Px is the only salivary enzyme produced exclusively in the salivary glands [39,55]. Thus, a decrease in Px activity may reflect the reduced activity of NWS in preventing free radical damage. Additionally, it is believed that in physiological concentrations, the main role in neutralizing hydrogen peroxide is played by Px, whereas in the conditions of ROS overproduction (extremely high levels of H_2_O_2_) this role is taken over by CAT [50,56]. It is therefore not surprising that we observed decreased Px activity in NWS, significantly decreased GPx activity in erythrocytes, and increased CAT activity in NWS of HF patients compared to healthy subjects.

The most important salivary antioxidant is UA, which accounts for up to 70%–80% of the antioxidant capacity of saliva [53,57]. The results of our study indicated an increased release of UA into HF patients’ saliva (compared to the controls), additionally raised with the progression of the disease (significantly higher UA concentration in NYHA III group vs. NYHA II). Uric acid is formed with the participation of xanthine oxidase (XO) that transforms xanthine into hypoxanthine. The process also generates free oxygen and nitrogen radicals [58,59]. HF hypoperfusion and tissue hypoxia secondarily activate XO and are thus responsible for oxidative and nitrosative cell damage [54,60]. Interestingly, boosted XO activity increases the expression of extracellular matrix metalloproteinases, which constitutes an important factor involved in myocardial post-infarction remodeling [61,62]. These enzymes have damaging effects on the vascular endothelium, but may also disturb the remodeling of the extracellular matrix of salivary glands [63]. Thus, when in high concentrations, UA has a prooxidative effect. It was demonstrated that this compound not only generates ROS, but also produces intermediate compounds capable of alkylation of biomolecules [58,59,64]. Moreover, hyperuricemia reduces the production of nitric oxide (NO), which leads to endothelial dysfunction. It is said that oxidative stress induced by hyperuricemia is also a factor promoting the development of insulin resistance [65].

In light of the above, it is obvious that in HF patients, we observed a significant decrease in TAC (in NWS, SWS and plasma alike) compared to the control. This parameter represents the total ability to sweep free radicals [42]. Thus, decreased TAC levels suggest the exhaustion of antioxidant reserves in HF patients. However, thiol groups also have a considerable share in the antioxidant activity of saliva and blood (besides UA), which may be indirectly indicated by decreased concentration of GSH (in NWS, SWS, and plasma) in the study group.

Decreased efficiency of the antioxidant barrier increases the risk of oxidative damage to cell components. This was confirmed by raised levels of TOS and OSI, as well as significantly higher oxidative damage to proteins (↑AGE, ↑AOPP) and lipids (↑MDA) in the saliva and plasma of HF patients compared to the controls; and the negative correlation between GSH concentration in NWS as well as SWS and AGE and AOPP concentration. Interestingly, ROS production, protein glycation, and peroxidation of salivary/plasma lipids increased considerably with the progression of heart failure (higher content of AGE and MDA and increased rate of ROS production in NYHA III vs. NYHA II). This indicates the deepening of redox homeostasis disorders together with the severity/degree of disease progression.

A very common ailment in patients with HF is disturbed saliva secretion (xerostomia and hyposalivation). This was also confirmed by the results of our study. In HF patients, we observed impaired secretory function of salivary glands, as evidenced by decreased salivary secretion, significantly lower total protein content, and lowered activity of α-amylase in NWS and SWS compared to the control. A negative correlation between NWS salivary flow and AGE and MDA content, as well as a positive correlation between ROS production rate in SWS and AOPP concentration in HF patients may suggest the effect of oxidative stress on salivary gland dysfunction. It can be assumed that protein/lipid oxidation products are aggregated and accumulated in salivary glands, which—on the basis of positive feedback—boosts ROS production and leads to further oxidation of biomolecules [16,25,66]. In the end, the secretory cells of salivary glands are damaged, which is manifested by decreased production of NWS and SWS as well as disturbances in protein synthesis/secretion to saliva. Interestingly, protein oxidation products (especially AGE) also can increase the production of pro-inflammatory cytokines, which enhances damage to the salivary glands [16,25]. The impairment of salivary gland function in HF patients is evidenced not only by a decrease in salivary flow rate but also by a reduction in salivary amylase activity and total protein content. Indeed, salivary amylase is the most important salivary enzyme that has been widely recognized as a marker of salivary hypofunction [67]. Although the salivary flow rate was significantly reduced for both non-stimulated and stimulated saliva, changes in α-amylase activity as well as disturbed protein secretion to saliva were found only in NWS of HF patients. In resting conditions, 80% of saliva is secreted by submandibular glands, while in stimulation, this activity is taken over by parotid glands [29,55]. Therefore, in patients with HF, mainly submandibular dysfunction occurs. Importantly, hyposalivation can result in oral mucositis, burning of the mouth, chewing and swallowing disorders, as well as speech disorders [67]. However, the volume of saliva may also be affected by cardiovascular drugs. Medicines that reduce saliva secretion include thiazide diuretics, beta-blockers, calcium channel blockers, and ACE inhibitors [3,68].

Changes in the qualitative and quantitative composition of saliva may also predispose to dental caries and periodontal disease [67]. Therefore, patients with HF should receive an additional dental care. Nevertheless, an open question is the possibility of antioxidant supplementation to improve the condition of HF cases. Due to the high clinical relevance, this issue requires further research and observations.

An important part of our study was also the assessment of diagnostic utility of redox biomarkers. We used ROC analysis to demonstrate that numerous parameters with high sensitivity and specificity differentiate subjects with NYHA II vs. NYHA III. Particularly promising results were observed for salivary AGE (AUC = 0.93, sensitivity = 81.82%, specificity = 82.35%, NYHA II vs. NYHA III), which was additionally confirmed by a positive correlation between salivary AGE content and blood NT-proBNP concentration, as well as a negative correlation with ejection fraction. Furthermore, the AGE concentration in NWS correlated positively with their plasma content. Therefore, saliva is an alternative biological material to blood, and its collection is cheap, non-invasive, and painless. Our experiment is the first to evaluate salivary redox homeostasis in HF patients, therefore, further studies are required to evaluate the clinical value of salivary redox biomarkers in a larger group of patients.

Despite the restrictive criteria of inclusion and exclusion, our study involved elderly people diagnosed, apart from HF, with type 2 diabetes, hypercholesterolemia, or hypertension. Thus, when characterizing the redox balance of our patients, we must consider not only heart failure, but also the accompanying diseases as well as the aging process. Similarly, in human studies, the oxidative stress caused by chronic vascular disease (CVD) versus HFrEF cannot be differentiated. HF is more common in men [69,70,71]; however, the unequal gender distribution in our study may be due that women reach a similar HF risk as men only after the menopause [3]. In addition, we evaluated only the selected oxidative stress biomarkers, which is also a limitation of the study. The exact effect of cardiological drugs on redox parameters is also unknown.

In conclusion, in HF patients, disturbances in enzymatic and non-enzymatic antioxidant defense as well as oxidative damage to proteins and lipids occur in NWS, SWS, and plasma/erythrocytes. Redox homeostasis disorders generally worsen with HF progression, and some parameters of oxidative stress in saliva may be potential diagnostic biomarkers. In patients with HF, mainly submandibular salivary glands are affected. Due to the qualitative and quantitative changes in saliva, HF patients should be provided with additional dental care.

## Figures and Tables

**Table 1:** Clinical characteristics of chronic heart failure (HF) patients and the control group.

Patient Characteristics		Control *n* = 50	NYHA II *n* = 33	NYHA III *n* = 17	ANOVA *p*
Gender	Male *n* (%)	43 (86)	29 (87.88)	14 (82.35)	NA
	Female *n* (%)	7 (14)	4 (12.12)	3 (17.65)	NA
Age		67.57 ± 1.11	64.2 ± 1.69	69.35 ± 2.57	0.069
WBC (x 10^3^/µL)		7.4 ± 0.16	7.25 ± 0.32	8.25 ± 0.46	0.068
RBC (x 10^6^/µL)		4.5 ± 0.06	4.72 ± 0.30	4.41 ± 0.13	0.573
HGB (g/dL)		14.07 ± 0.36	13.41 ± 0.27	13.4 ± 0.34	0.494
HCT (%)		39 ± 0.43	39.77± 0.78	39.04 ± 0.99	0.552
MCV (fL)		91.2 ± 0.60	90.97 ± 1.15	88.86 ± 1.37	0.373
MCH (pg)		33.62 ± 0.34	30.73 ± 0.50	29.69 ± 0.56 *	<0.001
MCHC (g/dL)		34.5 ± 0.36	33.75 ± 0.96	33.39 ± 0.56	0.059
RDW-SD (fL)		45.54 ± 0.16	46.56 ± 0.94	47.52 ± 0.95	0.091
RDV-CV (%)		14.9 ± 0.19	13.99 ± 0.27	14.71 ± 0.44	0.066
PLT (x10^3^/µL)		249 ± 1.79	187.4 ± 7.84 *	203.7 ± 15.44	<0.001
CRP (mg/L)		2.9 ± 0.2	3.2 ± 0.64	3.5 ± 0.85	0.308
Na^+^ (mmol/L)		139.2 ± 0.62	138.5 ± 0.45	136.6 ± 0.85	0.229
K^+^ (mmol/L)		4.2 ± 0.1	4.67 ± 0.10	4.74 ± 0.14	0.06
Creatinine (mg/dL)		0.9 ± 0.02	1.07 ± 0.06	1.32 ± 0.06	0.051
GFR (ml/min)		86. 03 ± 2.44	82.92 ± 0.59	72.33 ± 0.79	0.057
TSH (µIU/mL)		1.08 ± 0.03	1.03 ± 0.13	1.38 ± 0.30	0.06
FT3 (pg/mL)		2.31 ± 0.06	2.42 ± 0.11	2.29 ± 0.10	0.315
Vit. D_3_ (ng/mL)		24.3 ± 0.69	18.16 ± 1.41 *	15.07 ± 2.17 *	<0.001
AST (IU/L)		20.04 ± 0.87	23.46 ± 1.36	23.8 ± 1.83	0.06
Glucose (mg/dL)		91.5 ± 2.5	99.96 ± 3.83	99.8 ± 2.35	0.08
NT-proBNP (pg/mL)		ND	1831 ± 173	4128 ± 382	<0.001 ^#^
EF		ND	25.9 ± 1.24	19.24 ± 1.31	0.001 ^#^
RR (mmHg)	SBP	125.3 ± 0.33	130.6 ± 3.81	123.2 ± 3.67	0.112
	DBP	71.3 ± 0.61	75.97 ± 2.16	74.94 ± 1.99	0.06
HR		ND	70.97 ± 2.20	75.94 ± 2.59	NA
Type 2 diabetes *n* (%)		6 (12)	2 (11.76)	4 (23.53)	NA
Cardiac dysrhythmia (atrial flutter and fibrillation) *n* (%)		-	10 (30.30)	10 (58.82)	NA
Coronary artery disease *n* (%)		-	14 (42.42)	6 (35.29)	NA
Hypercholesterolemia *n* (%)		-	22 (66.67)	13 (76.47)	NA
Myocardial infarction *n* (%)		-	5 (15.15)	1 (5.88)	NA
Hypertension *n* (%)		17 (34)	26 (78.79)	12 (70.59)	NA
Medications	ASA *n* (%)	4 (8)	16 (48.48)	6 (35.29)	NA
	Alpha receptor blocker *n* (%)	-	3 (9.09)	2 (11.76)	NA
	Beta receptor blocker *n* (%)	6 (12)	26 (78.79)	15 (88.24)	NA
	Ca^2+^ channel blocker *n* (%)	4 (8)	9 (27.27)	6 (35.29)	NA
	AT1- receptor blocker *n* (%)	-	7 (21.21)	8 (47.1)	NA
	Diuretics *n* (%)	10 (20)	27 (81.82)	17 (100)	NA
	ACE *n* (%)	7 (14)	19 (57.58)	9 (52.94)	NA
	Cardiac glycosides *n* (%)	-	4 (12.12)	2 (11.76)	NA
	Organic nitrate *n* (%)	-	1 (3.03)	0 (0)	NA
	Statins *n* (%)	8 (16)	19 (57.58)	9 (52.94)	NA

Abbreviations: ACE—angiotensyn converting enzyme; ALT—alanine transferase; ASA—acetylsalicylic acid; AST—aspartate aminotransferase; CRP—c-reactive protein; DBP—diastolic blood pressure; EF—ejection fraction; FT3—free fraction of triiodothyronine; FT4—free fraction of thyroxine; GFR—glomerular filtration rate; HCT—hematocrit; HGB—hemoglobin concentration; HR—heart rate; K—potassium; MCH—mean corpuscular hemoglobin; MCHC—mean corpuscular hemoglobin concentration MCV—mean corpuscular volume; MPV— mean platelet volume; Na—sodium; NT-proBNP—N-terminal fragment of prohormone B-type natriuretic peptide; NWS—non-stimulated whole saliva; NYHA II—class II in the New York Heart Association (NYHA) classification of the heart failure; NYHA III—class III in the New York Heart Association (NYHA) classification of the heart failure; PCT—procalcitonin; PDW—platelet distribution width; P-LCR—platelet large cell ratio; PLT – platelets; RBC – red blood cells; RDW-CV – red cell distribution width, coefficient of variation; RDW-SD—red cell distribution width, standard deviation; RR—blood pressure; SBP—systolic blood pressure; TSH—thyroid stimulating hormone; WBC—white blood cells. *p ˂ 0.05 vs. the control group (Tukey’s test); ^#^*p* ˂ 0.05 vs. NYHA II (Student’s t-test).

**Table 2:** Salivary gland function and stomatological characteristics of HF patients and control subjects.

Patient Characteristics	Control *n* = 50	NYHA II *n* = 33	NYHA III *n* = 17	ANOVA *p*
NWS flow rate (mL/min)	0.38 ± 0.01	0.27 ± 0.02 *	0.25 ± 0.02 *	<0.001
SWS flow rate (mL/min)	1.28 ± 0.02	0.79 ± 0.07 *	0.76 ± 0.07 *	<0.001
NWS total protein (μg/mL)	1352 ± 62.73	1094 ± 63.84 *	8865 ± 67.08 *	<0.001
SWS total protein (μg/mL)	1014 ± 57.36	1023 ± 56.12	1309 ± 142.8	0.09
Salivary amylase NWS (µmol/mg protein)	0.2 ± 0.01	0.08 ± 0.01 *	0.13 ± 0.01 *	<0.001
Salivary amylase SWS (µmol/mg protein)	0.27 ± 0.02	0.17 ± 0.01 *	0.18 ± 0.01	*0.01*
DMFT	29.32 ± 0.53	29.96 ± 0.89	30.09 ± 0.69	NS
PBI	2.09 ± 0.07	2.12 ± 0.27	2.07 ± 0.24	0.92
GI	2.05 ± 0.06	2.06 ± 0.15	2.18 ± 0.14	0.71
CR	0.78 ± 0.73	0.63 ± 0.1	0.7 ± 0.48	0.91

Abbreviations: CR—root caries; DMFT—decayed, missing, filled teeth index; GI—gingival index; n—number of patients; NWS—non-stimulated saliva; NYHA II—class II in the New York Heart Association (NYHA) classification of the heart failure; NYHA III—class III in the New York Heart Association (NYHA) classification of the heart failure; PBI—papilla bleeding index; SWS—stimulated saliva. * *p* ˂ 0.05 vs. the control group.

**Table 3:** Receiver operating characteristic (ROC) analysis of oxidative stress biomarkers in non-stimulated and stimulated saliva as well as plasma/erythrocytes of NYHA II and NYHA III patients.

			NWS					SWS						Plasma/Erythrocytes				
Parameter	AUC	95% Confidence Interval	*p* Value	Cutt-off	Sensitivity (%)	Specificity (%)	AUC	95% Confidence Interval	*p* Value	Cut-off	Sensitivity (%)	Specificity (%)	AUC	95% Confidence Interval	*p* Value	Cut-off	Sensitivity (%)	Specificity (%)
*Antioxidants*																		
SOD (mU/mg protein)	0.5526	0.3788 to 0.7263	0.5457	> 5.616	57.58	58.82	0.6952	0.5457 to 0.8446	0.0249	> 7.742	57.58	58.82	0.5544	0.3678 to 0.7409	0.5322	> 0.6417	57.58	58.82
CAT (nmol H_2_O_2_/min/mg protein)	0.6061	0.4246 to 0.7875	0.223	< 0.72	57.58	58.82	0.6894	0.5316 to 0.8472	0.033	> 0.4727	63.64	62.5	0.6176	0.4517 to 0.7836	0.1765	> 0.3321	57.58	58.82
Px/GPx (mU/mg protein)	0.5686	0.4024 to 0.7349	0.4304	< 0.2229	57.58	58.82	0.5561	0.3737 to 0.7386	0.5189	> 0.4433	57.58	58.82	0.6791	0.5257 to 0.8325	0.0396	< 0.1417	63.64	64.71
GSH (ug/mg protein)	0.5918	0.4334 to 0.7502	0.2916	< 0.8745	57.58	58.82	0.574	0.4062 to 0.7417	0.3954	> 0.8199	57.58	58.82	0.6239	0.4368 to 0.8109	0.1546	< 3.37	57.58	58.82
UA (ng/mg protein)	0.7219	0.5818 to 0.862	0.0108	< 88.53	69.7	70.59	0.5152	0.34 to 0.6903	0.8618	> 104.2	42.42	41.18	0.852	0.748 to 0.9561	<0.0001	< 0.7263	75.76	76.47
*Total antioxidant/oxidant status*																		
TAC (Trolox umol/mg protein)	0.6667	0.4881 to 0.8453	0.0555	< 0.5182	57.58	58.82	0.6275	0.4644 to 0.7905	0.1431	> 0.3163	63.64	64.71	0.5686	0.3907 to 0.7465	0.4304	> 1.475	51.52	58.82
TOS (nmol H_2_O_2_/min/mg protein)	0.6916	0.5459 to 0.8373	0.0277	< 58.8	57.58	58.82	0.5152	0.3393 to 0.691	0.8618	< 61.38	48.48	47.06	0.5205	0.354 to 0.687	0.8138	> 15.14	57.58	58.82
OSI (TOS/TAC ratio)	0.5312	0.358 to 0.7044	0.72	> 132.6	51.52	52.94	0.6078	0.435 to 0.7807	0.2153	< 195.3	57.58	58.82	0.5116	0.3448 to 0.6784	0.8941	> 10.03	51.52	52.94
ROS production (nmol O_2-_/min/mg protein	0.877	0.7811 to 0.9729	<0.0001	< 14.49	81.82	82.35	0.5526	0.3832 to 0.7219	0.5457	> 15.96	54.55	52.94	ND	ND	ND	ND	ND	ND
*Oxidative damage products*																		
AGE (AFU/mg protein)	0.9251	0.8565 to 0.9937	<0.0001	< 9.3	81.82	82.35	0.5936	0.4192 to 0.7679	0.2823	> 8.173	54.55	52.94	0.9287	0.8582 to 0.9992	˂0.0001	< 7.028	81.82	82.35
AOPP (nmol/mg protein)	0.7879	0.653 to 0.9228	0.0009	< 52.89	69.7	70.59	0.8948	0.8039 to 0.9858	<0.0001	< 38.69	81.82	82.35	0.5294	0.3548 to 0.704	0.7354	> 7.937	57.58	58.82
MDA (umol/mg protein)	0.8699	0.7727 to 0.9671	<0.0001	< 349.1	78.79	76.47	0.5722	0.4005 to 0.7439	0.4069	> 651.3	54.55	52.94	0.9804	0.9491 to 1.012	˂0.0001	< 498.8	93.94	94.12

Abbreviations: AGE—advanced glycation end products; AOPP—advanced oxidation protein products; CAT—catalase; GPx—glutathione peroxidase; GSH—reduced glutathione; MDA—malondialdehyde; NWS—non-stimulated whole saliva; NYHA II—class II in the New York Heart Association (NYHA) classification of the heart failure; NYHA III—class III in the New York Heart Association (NYHA) classification of the heart failure; OSI—oxidative stress index; Px—salivary peroxidase; ROS—reactive oxygen species; SOD—superoxide dismutase; SWS—stimulated whole saliva; TAC—total antioxidant capacity; TOS—total oxidant status; UA—uric acid. Differences statistically important at: *p* < 0.05.
